# Fertility preservation in females requiring gonadotoxic therapy should be more than freezing measures before therapy – secondary fertility preservation and menopause care management after therapy should also be considered

**DOI:** 10.1007/s00404-025-08104-5

**Published:** 2025-07-12

**Authors:** Michael von Wolff, Sara Imboden, Petra Stute

**Affiliations:** 1 Division of Gynaecological Endocrinology and Reproductive Medicine, University Women’s Hospital, Inselspital, Friedbühlstrasse 19, 3010 Bern, Switzerland; 2 Department of Pelvic Cancer, Unit of Gynecologic Oncology, Karolinska University Hospital, Stockholm, Sweden

**Keywords:** Fertility preservation, Cancer, Primary fertility preservation, Secondary fertility preservation, Menopause, Oocyte freezing

## Abstract

To date, fertility preservation has mainly been offered to patients prior to gonadotoxic treatment. Ovarian reserve is assessed by analysing blood levels of anti-müllerian hormone (AMH), and gonadal cells or tissue are cryopreserved if indicated and requested by the patient. If primary fertility preservation (Primary FertiProtekt) before gonadotoxic treatment was not performed or was ineffective, secondary fertility preservation should be considered approximately one year after treatment based on a more extensive ovarian reserve analysis including menstrual cycle pattern, antral follicle count, and serum levels of AMH, estradiol and follicle stimulating hormone. Ovarian reserve analysis is also required to consider endocrine treatment in (pre) menopausal patients. Both approaches require the fertility preservation treatment to be tailored to the ovarian reserve status, type of gonadotoxic therapy. For secondary fertility preservation (Secondary FertiProtekt), oocyte freezing may be considered if ovarian reserve is not too low. Monthly treatment cycles, natural cycle or minimal stimulation protocols and follicle aspiration without anesthesia should be preferred. Menopause care management (MenoProtekt) involves acute menopausal symptom relief and prevention of chronic non-communicable diseases. The management needs to be individualized based on type of disease (hormone-dependent or -independent).

Fertility preservation before gonadotoxic therapy, especially in cancer, has been established almost worldwide for the last 20 years. It was initiated by the successful introduction of ovarian tissue freezing [1] and oocyte vitrification [2]. These new technologies set the tone for the emerging field of fertility preservation, which focussed mainly on freezing of gametes and gonadal tissue prior to gonadotoxic treatment. The success of these technologies, combined with the establishment of effective network structures [3], has indeed been very convincing, as effective fertility preservation can now be offered to most patients at need, with a chance of live birth of around 30% using the frozen material [2, 4].

However, in spite of these success rates, current fertility preservation programmes have limitations and carry some risks.

First, although a success rate of 30% per woman is a good success rate, it also means that in 70% of women it will be unsuccessful. Second, as fertility preservation cannot always be performed for medical and logistical reasons, a fertility reserve cannot be created in all women. Third, fertility preservation in children and adolescents is controversial because of the low risk of premature ovarian failure [5]. Fourth, because of the difficulty in defining the gonadotoxicity of treatments [6] and thereby the need for fertility preservation, some women are undertreated, whilst others are overtreated, with health risks due to postponement of cancer therapies and invasive fertility preservation measures.

The current concept of fertility preservation should therefore be modified. Fertility preservation should not only focus on freezing measures before gonadotoxic therapy (primary fertility preservation) as still recommend [7, 8] but should also include treatment after gonadotoxic therapy (secondary fertility preservation) and include endocrine and menopausal management (Fig. 1).

**Fig. 1:**
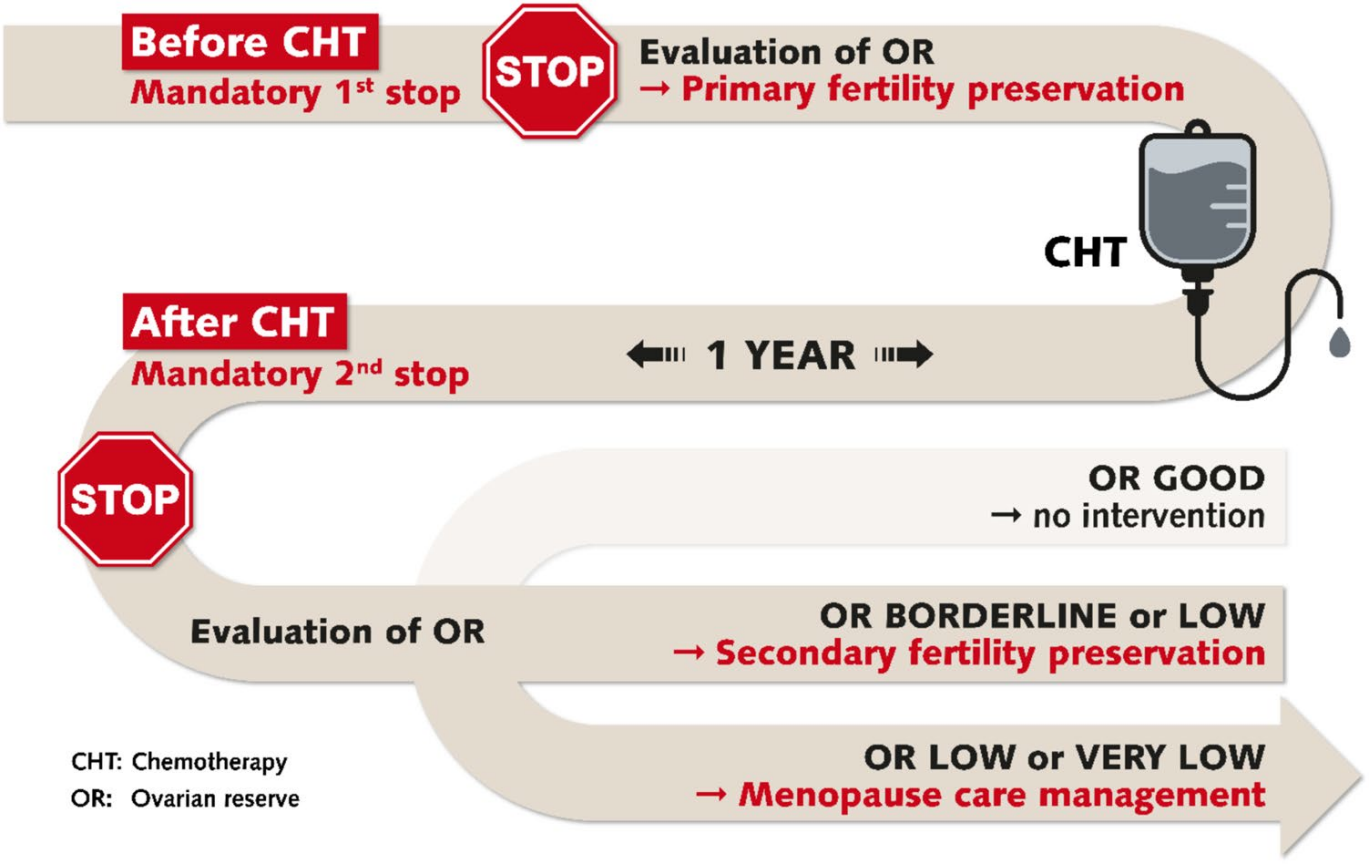
The suggested patient’s treatment journey of fertility preservation and endocrine/menopausal care

The patient’s treatment journey should still start with a mandatory first stop before gonadotoxic therapy to assess fertility by analysing anti-mullerian hormone (AMH) levels to estimate ovarian reserve. Women should be counselled on fertility issues and in case of therapies with proven gonadotoxicity, primary fertility preservation should be performed if medically and logistically possible.

During gonadotoxic treatment, endocrine treatment may be required to control heavy uterine bleeding, either by preventive treatment with GnRH agonists or by endocrine treatment with estradiol or progestagens.

After gonadotoxic treatment and a recovery period of about one year, a second mandatory stop is needed. During this second stop, ovarian function is again analysed by assessing AMH levels, as well as serum follicle-stimulating hormone (FSH) and estradiol (E2) concentrations and menstrual cycle patterns. Based on this assessment, women are divided into three ovarian reserve groups:

Group 1: Good ovarian reserve with normal or only slightly reduced AMH levels; Group 2: Borderline/low ovarian reserve with AMH levels just above the lower detection limit and still regular menstrual cycles; Group 3: Very low ovarian reserve and premature ovarian insufficiency (POI) with undetectable AMH concentration, elevated serum FSH concentration and oligo/amenorrhoea.

In cases with good ovarian reserve (group 1) and when cancer treatment is complete, no interventions are required. In cases with borderline/low ovarian reserve (group 2), secondary fertility preservation should be discussed. In cases with low/very low ovarian function (groups 2, 3), menopausal care management should also be considered (Fig. 1).

Secondary fertility preservation requires different measures than primary fertility preservation. Ovarian tissue freezing is recommended in cases such as leukaemia, when patients have not undergone primary fertility preservation and are facing highly gonadotoxic bone marrow transplantation. Ovarian reserve is still sufficiently high and induction chemotherapy does not interfere with ovarian tissue freezing [9]. Oocyte freezing is recommended for women with regular cycles and borderline ovarian reserve. In contrast to full gonadotropin stimulation prior to cancer therapy, reduced ovarian reserve requires consecutive and monthly treatment cycles with low-dose gonadotropin stimulation, possibly in combination with oral agents such as letrozole or clomiphene citrate, or, in the case of low or very low, ovarian reserve, natural cycle approaches to achieve highest and most cost-effective therapy [10].

If ovarian reserve is low (group 2) or very low / POI (group 3), patients should also be referred to a menopause specialist, as untreated POI is a significant health risk [11].

In general, management should follow current POI guidelines, which include systemic hormone therapy (HT) at standard doses of oestrogens with or without progestins. In women with systemic hormone-dependent malignancies, several non-hormonal treatment options are available, such as the NK3R antagonist fezolinetant, antidepressants, or stellate ganglion block [12]. For the management of the genitourinary syndrome of menopause low-dose vaginal estrogens or vaginal prasterone may be considered if the patient does not respond to non-hormonal treatments or vaginal laser therapy [13]. In addition, bone [14] and cardiovascular [15] health in cancer patients should be assessed, monitored and managed according to appropriate guidelines.

The strength of the strategy described in this paper is obvious. Fertility preservation is more individualised, unnecessary preservation measures and risks are reduced and the efficacy of preservation measures, i.e. the chance to conceive with the frozen material can be increased if primary and secondary preservation is combined.

However, the strategy also imposes a risk. If oncologists, reproductive physicians and patients rely too much on the option of secondary fertility preservation, fertility preservation before gonadotoxic therapy might be skipped. If the patient has developed a POI or a very low ovarian reserve, she will not be able to freeze any gametes or ovarian tissue anymore. Therefore, patients should still be counselled by competent reproductive physicians with a high expertise to decide which type of fertility preservation is the best, primary, secondary or possibly the combination of both strategies.

In conclusion, fertility, endocrine and menopausal management need to be addressed not only before but also after gonadotoxic therapy. Therefore, a second stop approach approximately one year after completion of gonadotoxic therapy is strongly recommended and need to be introduced to assess ovarian reserve and, if needed, to provide secondary fertility preservation or menopausal management. Such an approach will require adjustments to fertility preservation and reimbursement programmes, but will improve fertility and health care and may also save costs by reducing unnecessary fertility preservation measures prior to cancer treatment.

## Data Availability

No datasets were generated or analysed during the current study.
